# How do implants overlying the spine influence “The Law of Diminishing Returns” in early-onset scoliosis patients?

**DOI:** 10.1007/s43390-024-00885-0

**Published:** 2024-05-08

**Authors:** Stuart L. Mitchell, Jessica H. Heyer, Jason B. Anari, Keith D. Baldwin, Pranav Kodali, Brandon S. Ramo, Jack M. Flynn, Ryan Fitzgerald, Walter Truong, Ying Li, Lindsay Andras, Jaysson Brooks, Patrick J. Cahill

**Affiliations:** 1 Department of Orthopaedics, University of North Carolina, Chapel Hill, NC USA; 2 Department of Pediatric Orthopaedics, Hospital for Special Surgery, New York, NY USA; 3 Department of Orthopaedics, The Children’s Hospital of Philadelphia, Philadelphia, PA USA; 4 University of Pennsylvania Perelman School of Medicine, Philadelphia, PA USA; 5 Department of Orthopaedic Surgery, University of Texas Southwestern Medical Center, Scottish Rite for Children, Dallas, TX USA; 6 Children’s Orthopaedic and Scoliosis Surgery Associates, St. Petersburg, FL USA; 7 Department of Orthopaedics, Gillette Children’s, St. Paul, MN USA; 8 Department of Orthopaedics, University of Michigan, Michigan Medicine, Ann Arbor, MI USA; 9 Department of Orthopaedics, Children’s Hospital of Los Angeles, Los Angeles, CA USA

**Keywords:** Early-onset scoliosis, Distraction-based spine implants, Growing rods, Pediatric spinal deformity

## Abstract

**Purpose:**

The “law of diminishing returns” (LODR) in early-onset scoliosis (EOS) is well-known. We hypothesized that previously observed variations between constructs may be related to the lateral distance that each construct lies from the spine. We therefore sought to determine whether the curve magnitude improvement and spinal length gains for distraction-based constructs in EOS are positively correlated with the collinearity of the spine and the convex-sided implant on posteroanterior radiographs.

**Methods:**

A prospectively-collected, multicenter EOS registry was queried for all patients who underwent non-fusion, distraction-based instrumentation surgery. Post-index radiographs were graded from 1 to 5 based on amount of overlap between the convex-sided rod and the apical vertebra. Grade 1: convex rod is lateral to convex-sided pedicle; Grade 2: overlaps the convex-sided pedicle; Grade 3: lies between pedicles; Grade 4: overlaps concave-sided pedicle; Grade 5: medial to concave-sided pedicle. ANOVA assessed the correlations between post-index overlap grade and change in (a) curve magnitude and (b) T1–T12 height. Multivariable regression modeling further assessed these associations.

**Results:**

284 patients met all selection criteria and were included. On ANOVA, post-index grade was associated with curve magnitude (p <0.001) and T1-12 height (p = 0.028) change. Better curve correction and height change were associated with higher grade. On regression modeling, curve correction (R = 0.574) and T1–T12 height change (R = 0.339) remained significantly associated with grade when controlling for time, anchor locations, age, underlying diagnosis, and pre-index curve magnitude.

**Conclusion:**

More apical overlap by the convex rod was associated with better spinal deformity control and improved height gain.

**Level of Evidence III:**

Therapeutic.

## Introduction

Early-onset scoliosis (EOS), defined as coronal curvature of the spine of 10 degrees or more in patients under ten years of age at the time of diagnosis [1, 2], is a heterogenous and complex problem to treat. At present, it is not always clear how best to manage certain EOS curves because physicians must balance continued spinal and thoracic growth with the need to control curves of worsening severity. Additionally, the patient population is heterogeneous, with a variety of underlying diagnoses, including the idiopathic population. Curves may be managed with observation alone, bracing, casting, or surgery. When curves progress in severity, distraction-based growth-friendly instrumentation surgery or growth guidance surgery (e.g., Shilla technique) may be recommended to decrease spine and chest wall deformity while permitting continued growth and lung development [3]. There are many implant choices available for pediatric spine surgeons for distraction-based instrumentation surgery that vary with regards to anchor points used (e.g., rib, spine, and pelvis) and the types of rods being used (e.g., traditional growing rods [TGR], vertical expandable prosthetic titanium rib [VEPTR], magnetically-controlled growing rods [MCGR], etc.).

Once distraction-based instrumentation surgery is undertaken, the race begins between distraction of the rods to maintain longitudinal growth of trunk and spine and spontaneous stiffening with reduced lengthening of the spine. This observation has been previously termed the “law of diminishing returns” (LODR) [4]. This LODR is well known and was first observed with traditional growing rod (TGRs) constructs [4, 5]. More frequent lengthening with magnetically-controlled growing rods (MCGRs) was theorized to potentially obviate autofusion with decreased “immobilization” time between lengthenings. However, data remains mixed for MCGR constructs [6–12]. More confusing still, investigators have demonstrated that this law of diminishing returns does not apply to rib-based distraction constructs [13], especially with more frequent lengthenings of less than every 7 months [14]. This difference between constructs may be related to the lateral distance that each construct lies from the spine [15].

To further elucidate the causes of the LODR, we sought to determine whether the curve magnitude and spinal length gains for growth-friendly spine constructs in EOS are correlated with the amount of overlap of the spine with the convex-sided implant on posteroanterior (PA) radiographs. We hypothesized that more overlap of the spine with the convex-sided implant (i.e., higher grade) would result in worse curve magnitude correction and worse spinal length (height) gain.

### Methods

A prospectively-collected multicenter registry of patients with early onset scoliosis was queried for all patients who underwent distraction-based instrumentation surgery. Institutional review board approval was obtained at each of the participating institutions that enroll patients into the database. We included only patients who were <10 years of age at index surgery with ≥3 years of follow-up, ≥3 lengthenings, and bilateral distraction-based spinal instrumentation constructs. Patients with an underlying diagnosis of idiopathic, neuromuscular, or syndromic scoliosis were eligible for inclusion. Patients who were missing data including post-index and pre-definitive fusion or final follow-up curve magnitude, T1–12 measurements, or radiographs and those who underwent index surgery with definitive posterior spinal fusion or guided growth techniques (e.g., Luqué trolley, Shilla technique, etc.) were excluded. Patients with an underlying diagnosis of skeletal dysplasia, congenital scoliosis, or thoracogenic scoliosis were also excluded. All remaining patients available in the databased meeting these selection criteria were included and analyzed.

**Fig 1:**
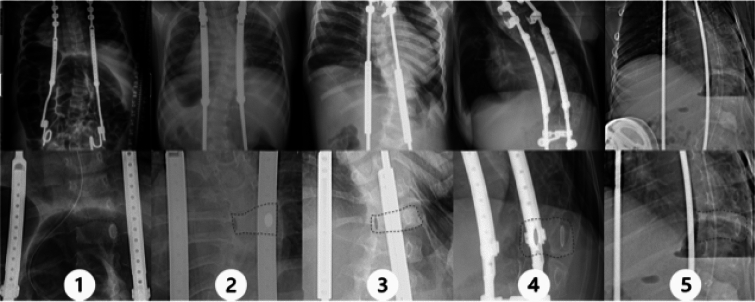
Radiographic appearance of example patients of grades 1–5 based on the location of the apical vertebra relative to the convex-sided spinal rod. The convex rod is lateral to the convex-sided pedicle for grade 1, overlaps the convex-sided pedicle for grade 2, lies between the pedicles for grade 3, overlaps the concave-sided pedicle for grade 4, and is medial to the concave-sided pedicle for grade 5

Post-index radiographs were reviewed and graded from 1 to 5 based on amount of implant overlap with the convex-sided rod at the apical vertebra (Fig. 1). All images were graded by a research assistant and attending pediatric orthopaedic surgeon and reviewed by a second pediatric orthopaedic surgeon if any disagreement. Grade 1 is defined as the convex rod being lateral to the convex-sided pedicle of the major curve; Grade 2, the convex rod overlaps the convex-sided pedicle; Grade 3, the convex rod lies between the pedicles; Grade 4, the convex rod overlaps the *concave*-sided pedicle; Grade 5, the convex rod is medial to the *concave*-sided pedicle.

Demographic data were summarized using descriptive statistics. Two-tailed, unpaired t-tests were used to compare means curve magnitude change of the major coronal curve and height change from post-index surgery to most recent/final follow-up. One-way ANOVA was performed to assess association between post-index grade and post-index minus pre-definitive/most recent curve magnitude, post-index grade and post-index minus pre-definitive/most recent T1–T12 height, and for post-index grade and post-index minus pre-definitive/most recent L1–S1 height. In cases where definitive fusion was performed, the curve magnitude or T1–T12 height from immediately prior to the fusion surgery was used, but in patients who did not undergo a final fusion, the curve magnitude or T1–T12 height from the most recent follow-up radiographs were used. Multivariable regression modeling was used further assess the association between post-index grade and curve magnitude correction or change in T1–T12 height. The multivariable models included either curve magnitude change, or T1–12 height change and post-index grade, anchor location (ribs, spine, pelvis), age at index surgery, underlying diagnosis, and time from index surgery to pre-definitive or final X-ray. Significance was set at a = 0.05. Analyses were performed using IBM SPSS Statistics for Mac (IBM, Armonk, NY).

## Results

**Fig 2:**
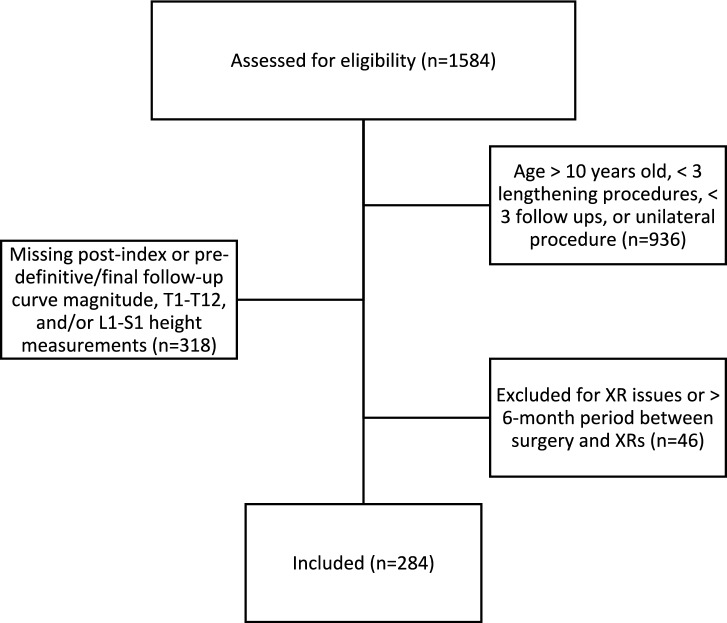
CONSORT diagram illustrating flow of patient selection

Of the initial 1584 patients identified in the query, only 284 patients (17.9%) met all selection criteria and were included (Fig. 2). The majority (54%) were female and had neuromuscular scoliosis (59%); patients were 6.7 ± 2.1-years-old at the time of index surgery with 7.1 ± 3.1 years of follow-up. Not all patients were followed until “graduation” but all were followed until final fusion, graduation, or until final follow-up available in the database. The average curve magnitude measurement of the major curve prior to the index distraction-based instrumentation surgery measured 72.0 ± 21.7 degrees (Table 1). Post-index radiographs were classified as grade 1 in 26 patients, (9%), grade 2 in 75 patients (26%), grade 3 (38%) in 107 patients, grade 4 in 56 patients (20%), and grade 5 in 20 patients (7%). For the overall cohort, we observed decreased curve magnitude immediately following index implant surgery with slight increase in curve magnitude over time (Table 1). With regards to spine length, initial improvements were observed with further length gains at the time of pre-definitive fusion or final follow-up.

**Table 1:** Demographic and surgical factors for 284 patients treated with posterior distraction-based instrumentation for early-onset scoliosis stratified by their post-index radiographic grade

	Grade 1 n=26	Grade 2 n=75	Grade 3 n=107	Grade 4 n=56	Grade 5 n=20	Total n=284
Female (%)	50	55	55	54	45	54
Age at index (yrs)	6.7±2.3	6.2±2.3	6.8±2.1	7.1±1.9	6.3±2.1	6.6±2.2
Diagnosis, n (%) Idiopathic Neuromuscular Syndromic	0 (0%) 23 (88%) 3 (12%)	5 (7%) 57 (76%) 13 (17%)	19 (18%) 53 (50%) 35 (33%)	11 (20%) 25 (45%) 20 (36%)	4 (9%) 10 (22%) 6 (13%)	39 (14%) 168 (59%) 77 (30%)
Time from index to final follow-up (yrs)	7.3±2.7	6.9±2.7	7.2±3.5	6.6±2.8	8.6±3.0	7.0±3.1
Index implant details						
Device used, n (%)						
*VEPTR*	22 (85%)	43 (57%)	25 (23%)	9 (16%)	3 (15%)	102 (36%)
*MCGR*	3 (12%)	18 (24%)	33 (31%)	17 (30%)	1 (5%)	72 (25%)
*TGR*	1 (4%)	14 (19%)	49 (46%)	30 (54%)	16 (80%)	110 (39%)
Proximal anchor location						
*Ribs*	25 (96%)	43 (57%)	27 (25%)	12 (21%)	3 (15%)	110 (39%)
*Spine*	0 (0%)	31 (41%)	79 (74%)	43 (77%)	17 (85%)	170 (60%)
*Spine+Rib(s)*	1 (4%)	1 (1%)	0 (0%)	1 (2%)	0 (0%)	3 (1%)
*Missing*	0 (0%)	0 (0%)	1 (1%)	0 (0%)	0 (0%)	1 (0%)
Distal anchor location						
*Rib*	1 (4%)	0 (0%)	0 (0%)	0 (0%)	0 (0%)	1 (0%)
*Spine*	1 (4%)	30 (40%)	71 (66%)	45 (80%)	13 (65%)	160 (56%)
*Pelvis*	24 (92%)	39 (52%)	23 (21%)	6 (11%)	3 (15%)	95 (33%)
*Pelvis+Spine*	0 (0%)	5 (7%)	11 (10%)	5 (9%)	4 (20%)	25 (9%)
*Missing*	0 (0%)	1 (1%)	2 (2%)	0 (0%)	0 (0%)	3 (1%)
Pre-index						
Curve magnitude (°)	62±24	64±20	71±19	84±18	90±21	72±22
T1–12 height (cm)	17.4±3.5	16.6±3.3	16.5±2.9	15.6±3.6	15.0±2.5	16.3±3.2
L1–S1 height (cm)	9.2±2.7	9.8±2.4	10.1±2.3	9.1±2.4	9.0±2.0	9.7±2.4
T1–S1 height (cm)	26.6±5.7	26.4±5.2	26.7±4.5	24.7±4.6	23.9±3.2	26.0±4.8
Post-index						
Curve magnitude (°)	32±18	39±17	42±14	53±15	61±19	44±17
T1–12 height (cm)	19.3±4.1	18.9±3.4	19.0±2.7	18.4±2.7	17.8±2.3	18.8±3.0
L1–S1 height (cm)	10.5±3.5	11.3±1.9	11.6±2.3	11.8±2.0	10.7±1.9	11.4±2.3
T1–S1 height (cm)	29.4±5.8	30.2±5.1	30.6±4.3	30.2±4.1	28.5±3.4	30.2±4.6
Pre-definitive/final follow-up						
Curve magnitude (°)	57±26	55±23	50±20	58±19	60±20	54±21
T1–12 height (cm)	21.2±4.6	20.4±4.4	21.9±3.3	20.7±3.6	21.7±3.2	21.2±3.8
L1–S1 height (cm)	12.5±3.5	12.2±2.4	13.1±2.3	12.7±2.5	12.0±1.6	12.6±2.4
T1–S1 height (cm)	33.8±7.8	32.5±6.1	35.0±4.8	33.4±4.9	33.8±4.1	33.8±5.5
Underwent final fusion	31%	29%	32%	43%	55%	35%

## Discussion

Severe early-onset scoliosis remains a challenging condition to treat. This study shows that following distraction-based spinal instrumentation surgery, there is an association with the amount of convex rod overlap with the spine and the curve magnitude correction and T1–T12 height change, but not with L1–S1 height change. However, our null hypothesis was accepted as we found that mean curve magnitude correction and mean T1–T12 height change were the opposite of what was expected (i.e., more overlap of the spine with the convex-sided implant resulted in worse curve magnitude correction and worse spinal length gain). Specifically, we found that lower grade was associated with worsening curve magnitude over time from post-index to final radiographs. The relationship between post-index grade and change in T1–T12 height was not as clear, but similarly, T1–T12 height improved less in patients with lower grade.

Many studies have sought to determine the causes of the “law of diminishing returns” observed with growth-friendly spinal implants in EOS and methods to avoid this detrimental effect [4, 6–11, 13, 14, 16]. Investigators have evaluated the anatomic location of anchors used, underlying etiology of scoliosis, implant type, implant size, body size, age, sagittal parameters, and many more. However, to our knowledge, no studies have directly evaluated the effect of the spatial relationship of the implant to the spine on the immediate post-index coronal radiographs in terms of predicting deformity control and the occurrence of the LODR. A recent study examined a related concept investigating the effect of apical vertebral position relative to growing rod position using a finite element analysis [17]. After index TGR or MCGR (spine and/or pelvis anchors only), patients were categorized as Groups 1–3 based on the location of the apical vertebra relative to the rods. In Group 1, both pedicles were located between the rods, in Group 2, the convex rod was between the pedicles, and in Group 3, both pedicles were lateral to the convex rod. In this more homogeneous patient cohort, they concluded that bringing the apex in line with the rods (Group 1) results in the largest height gain and deformity control.

**Fig 3:**
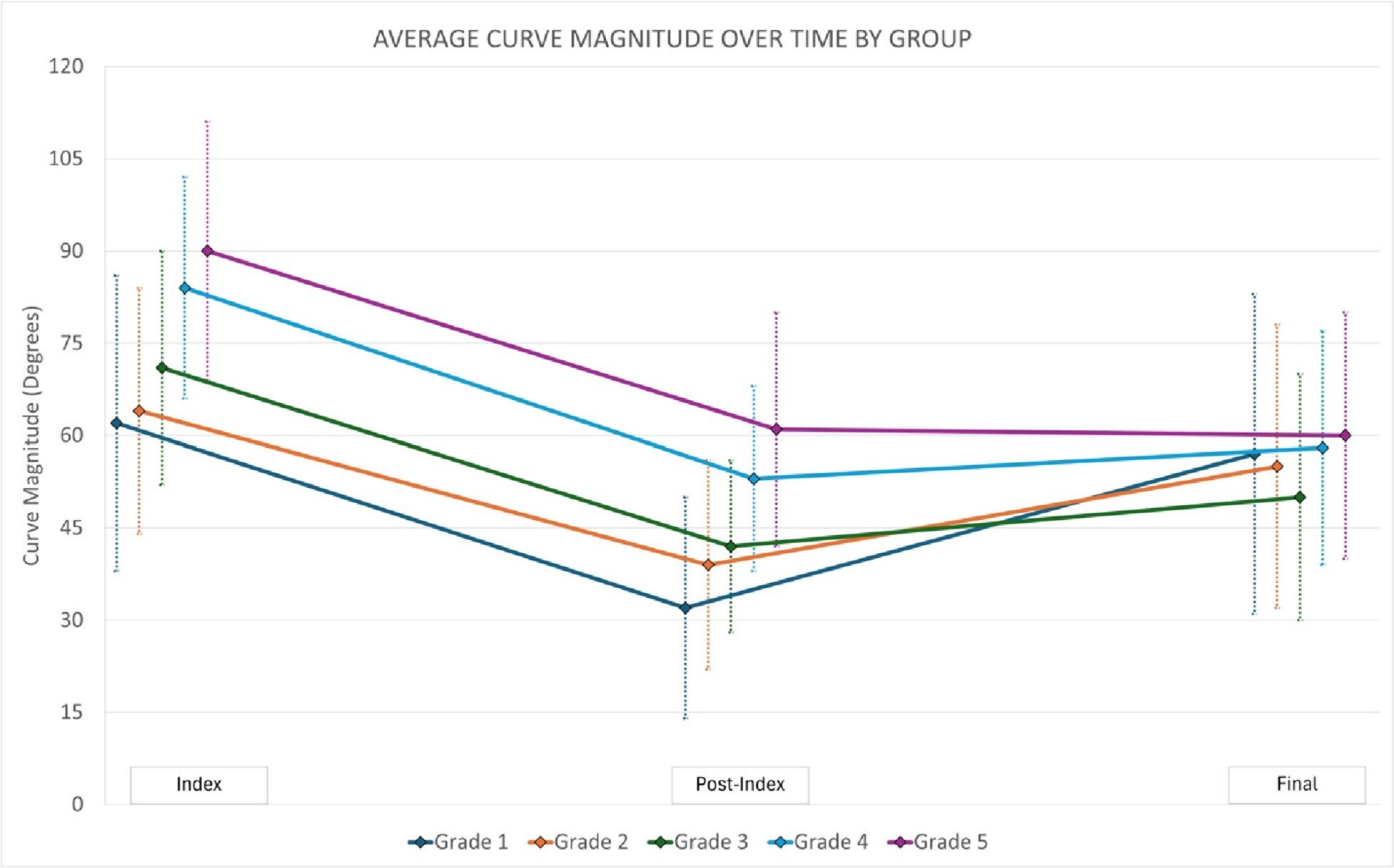
Average curve magnitude change from pre-index surgery to post-index surgery to final follow-up or pre-definitive surgery stratified by post-index grade of the amount of implant overlap at the curve apex. Please note that ‘final’ refers to final follow-up (if no final fusion) or prior to definitive fusion surgery

We were surprised that in our cohort more apical overlap by the convex rod (i.e., higher grade) is positively correlated with better control of the spinal deformity and improved height gain. This could potentially result from unintended apical control via the rod serving as a blocker to further lateral deviation and/or axial plane progression. Other investigators have specifically created constructs which capture the convex apical vertebra at 1-2 levels with screws with the goal of improving apical rotation [18]. Similarly, Shilla growth guidance constructs achieve a similar goal, but have had mixed results regarding deformity correction and height gain compared to use of TGRs or MCGRs [19, 20]. Alternatively, there may be unintended fusion adjacent to the rod on the convexity slowing growth relative to the concavity in the higher-grade patients. We noted that the average post-index and final curve magnitudes were essentially unchanged in grade 5 patients from 61° to 60°, respectively, suggesting improved control of the deformity, similar to Shilla technique. Curves were noted to be larger preoperatively in higher grade patients (average, 62° and 64° in grades 1 and 2, respectively, vs 84° and 90° in grades 4 and 5, respectively). Grade 1 and 2 patients with smaller curves preoperatively may have had all the “slack” removed from the system with distraction during implantation as reflected by their smaller post-index curve magnitudes (average, 32° and 39° in grades 1 and 2, respectively) compared to higher grade patients (average, 53° and 61° in grades 4 and 5, respectively). During subsequent lengthenings, all patients had improved height but not curve correction in the smaller curves whereas the curves that were larger pre-operatively in grade 4 and 5 patients continued to improve, or at least not worsen as rapidly as they had more “slack” left in the system to be removed. The patients with lower grade (e.g., grades 1 and 2) have already had the slack removed from the system so the smaller length gains are likely a reflection of only spinal growth as compared to spinal growth and additional removal of “slack” from the system in the higher-grade patients. Lastly, there was a much higher proportion of patients with VEPTR/rib anchors and pelvis anchors in grades 1 and 2 compared to higher grades. This may indicate that the rods are sitting further/more lateral from the spine but do not control the deformity as effectively. Conversely, there appear to be more spine-based anchors in the higher grade which may be an indication that the rods are starting in closer proximity to the spine for similar curve magnitude. Perhaps, the higher grade patients have more autofusion due to approach and rod location near the spine. We observed that their curves did not correct as much from post-index to final, but also aren’t worsening as much relatively as compared to the lower grade patients from post-index to final (Fig. 3). However, the concept of “protective apical autofusion” does not explain the larger increase in height in patients with a higher grade of implant overlap.

**Fig 4:**
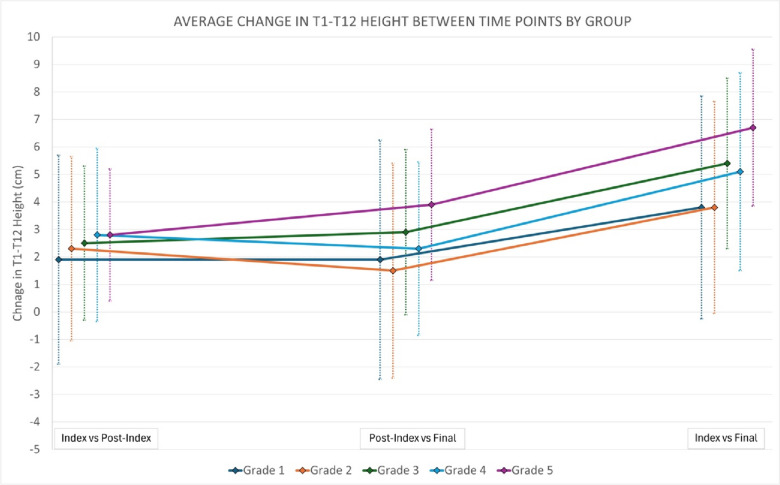
Average T1-T12 height change at different time points from pre- to post-index surgery, post-index to final, and from post-index to final stratified by post-index grade of the amount of implant overlap at the curve apex. Please note that ‘final’ refers to final follow-up (if no final fusion) or prior to definitive fusion surgery

It is important to note that while the experimental factor (i.e., grade) remains significantly positively correlated with the outcomes (i.e., major curve magnitude change and T1–T12 height change) it only accounted for about 33% of the variance in the curve magnitude change model and 12% of the variance of the T1–T12 height change model. Multiple important confounders were included in the models such as age, time of follow-up, pre-index major curve magnitude, underlying diagnosis, and anchor locations. Despite including these, 77% and 88% of the variance is not accounted for in the models. Based on review of the literature, the remaining variance could potentially be explained, at least in part, by a lack of apical control, body size, lengthening time interval, sagittal parameters, force applied in lengthening, or implant type used [9, 16–19, 21]. There is likely also a large degree of unexplained variance and thus further investigation is warranted to identify the source of this remaining variance Fig. 4.

As with any retrospective database analysis, this study has limitations. Although this is a prospectively managed database, there are still large amounts of data missing which led to the exclusion of many patients. However, this is an international multicenter database and thus greatly improves the generalizability of our findings. Grade 1 and grade 5 group sizes were relatively small, however, each group included at least 20 patients. Additional strengths of our study include the minimum follow-up of 3 years and the overall large sample size.

Based on our findings presented here, it appears that the degree of rod overlap with the spine on post-index radiographs is correlated with the amount of correction and growth achieved after distraction-based instrumentation surgery for early-onset scoliosis, but in the opposite direction as hypothesized. Specifically, more apical overlap by the convex rod (e.g., higher grade) was associated with better spinal deformity control and improved height gain. Rod location relative to the apex of the major curve in early-onset scoliosis likely contributes to the observed law of diminishing returns, but the implications are complex and only account for a small degree of the observed phenomenon.

## Declarations

**Ethical approval**

This study was approved by the Institutional Review Board, and informed consent was obtained from legal guardians.

## References

[1] El-Hawary R, Akbarnia BA (2015) Early onset scoliosis–time for consensus. Spine Deform 3(2):105–106. 10.1016/j.jspd.2015.01.003 27927299 10.1016/j.jspd.2015.01.003

[2] Skaggs DL, Guillaume T, El-Hawary R, Emans J, Mendelow M, Smith J (2015) Early onset scoliosis consensus statement, SRS Growing Spine Committee, 2015. Spine Deform 3(2):107 10.1016/j.jspd.2015.01.002

[3] Yang S, Andras LM, Redding GJ, Skaggs DL (2016) Early onset scoliosis: a review of history current treatment and future directions. Pediatrics 137:1 10.1542/peds.2015-0709 26644484

[4] Sankar WN, Skaggs DL, Yazici M et al (2011) Lengthening of dual growing rods and the law of diminishing returns. Spine 36(10):806–809. 10.1097/BRS.0b013e318214d78f 21336236 10.1097/BRS.0b013e318214d78f

[5] Cahill PJ, Marvil S, Cuddihy L et al (2010) Autofusion in the immature spine treated with growing rods. Spine 35(22):E1199-203. 10.1097/BRS.0b013e3181e21b50 20683383 10.1097/BRS.0b013e3181e21b50

[6] Ahmad A, Subramanian T, Panteliadis P, Wilson-Macdonald J, Rothenfluh DA, Nnadi C (2017) Quantifying the ‘law of diminishing returns’ in magnetically controlled growing rods. Bone Jt J 99B(12):1658–1664. 10.1302/0301-620X.99B12.BJJ-2017-0402.R2 10.1302/0301-620X.99B12.BJJ-2017-0402.R2 29212690

[7] Cheung JP, Bow C, Samartzis D, Kwan K, Cheung KM (2016) Frequent small distractions with a magnetically controlled growing rod for early-onset scoliosis and avoidance of the law of diminishing returns. J Orthop Surg (Hong Kong) 24(3):332–337. 10.1177/1602400312 28031501 10.1177/1602400312

[8] Cheung JPY, Bow C, Cheung KMC (2022) Law of temporary diminishing distraction gains’’: the phenomenon of temporary diminished distraction lengths with magnetically controlled growing rods that is reverted with rod exchange. Global Spine J 12(2):221–228. 10.1177/2192568220948475 32799681 10.1177/2192568220948475 PMC8907632

[9] Cheung JPY, Yiu KKL, Samartzis D, Kwan K, Tan BB, Cheung KMC (2018) Rod lengthening with the magnetically controlled growing rod: factors influencing rod slippage and reduced gains during distractions. Spine 43(7):E399–E405. 10.1097/BRS.0000000000002358 28767632 10.1097/BRS.0000000000002358

[10] Gardner A, Beaven A, Marks D, Spilsbury J, Mehta J, Newton Ede M (2017) Does the law of diminishing returns apply to the lengthening of the MCGR rod in early onset scoliosis with reference to growth velocity? J Spine Surg 3(4):525–530. 10.21037/jss.2017.08.16 29354727 10.21037/jss.2017.08.16 PMC5760426

[11] Heyer JH, Anari JB, Baldwin KD et al (2022) lengthening behavior of magnetically controlled growing rods in early-onset scoliosis: a multicenter study. JBJS 104(24):2186–2194 10.2106/JBJS.22.00483 36367763

[12] Lampe LP, Schulze Bovingloh A, Gosheger G, Schulte TL, Lange T (2019) Magnetically controlled growing rods in treatment of early-onset scoliosis: a single center study with a minimum of 2-year-follow up and preliminary results after converting surgery. Spine 44(17):1201–1210. 10.1097/BRS.0000000000003048 30985569 10.1097/BRS.0000000000003048

[13] El-Hawary R, Samdani A, Wade J et al (2016) Rib-based distraction surgery maintains total spine growth. J Pediatr Orthop 36(8):841–846. 10.1097/BPO.0000000000000567 26090967 10.1097/BPO.0000000000000567

[14] Qiu C, Lott C, Agaba P, Cahill PJ, Anari JB (2020) Lengthening less than 7 months leads to greater spinal height gain with rib-based distraction. J Pediatr Orthop 40(8):e747–e752. 10.1097/BPO.0000000000001625 32776773 10.1097/BPO.0000000000001625

[15] Heyer JH, Anari JB, Baldwin KD et al (2023) Rib-to-spine and rib-to-pelvis magnetically controlled growing rods: does the law of diminishing returns still apply? Spine Deform. 10.1007/s43390-023-00718-6 37450222 10.1007/s43390-023-00718-6

[16] Saarinen AJ, Sponseller PD, Andras LM et al (2022) Matched comparison of magnetically controlled growing rods with traditional growing rods in severe early-onset scoliosis of >/=90 degrees: an interim report on outcomes 2 years after treatment. J Bone Jt Surg Am 104(1):41–48. 10.2106/JBJS.20.02108 10.2106/JBJS.20.02108 34644282

[17] Dursun G, Cetik RM, Guzel D et al (2022) The effect of apical vertebra position on growing rod treatment: a clinical and finite element study. J Pediatr Orthop 42(6):e552–e558. 10.1097/BPO.0000000000002135 35297388 10.1097/BPO.0000000000002135

[18] Wijdicks SPJ, Skov ST, Li H, Castelein RM, Kruyt MC, Bunger C (2020) 3-Year follow-up of a single magnetically controlled growing rod with contralateral gliding system and apical control for early onset scoliosis. Spine Deform 8(4):751–761. 10.1007/s43390-020-00098-1 32232747 10.1007/s43390-020-00098-1 PMC7366570

[19] Andras LM, Joiner ER, McCarthy RE et al (2015) Growing rods versus shilla growth guidance: better cobb angle correction and T1–S1 length increase but more surgeries. Spine Deform 3(3):246–252. 10.1016/j.jspd.2014.11.005 27927466 10.1016/j.jspd.2014.11.005

[20] Haapala H, Saarinen AJ, Salonen A, Helenius I (2020) Shilla growth guidance compared with magnetically controlled growing rods in the treatment of neuromuscular and syndromic early-onset scoliosis. Spine 45(23):E1604–E1614. 10.1097/BRS.0000000000003654 32858743 10.1097/BRS.0000000000003654

[21] Spurway AJ, Chukwunyerenwa CK, Kishta WE, Hurry JK, El-Hawary R (2016) Sagittal spine length measurement: a novel technique to assess growth of the spine. Spine Deform 4(5):331–337. 10.1016/j.jspd.2016.03.002 27927489 10.1016/j.jspd.2016.03.002

